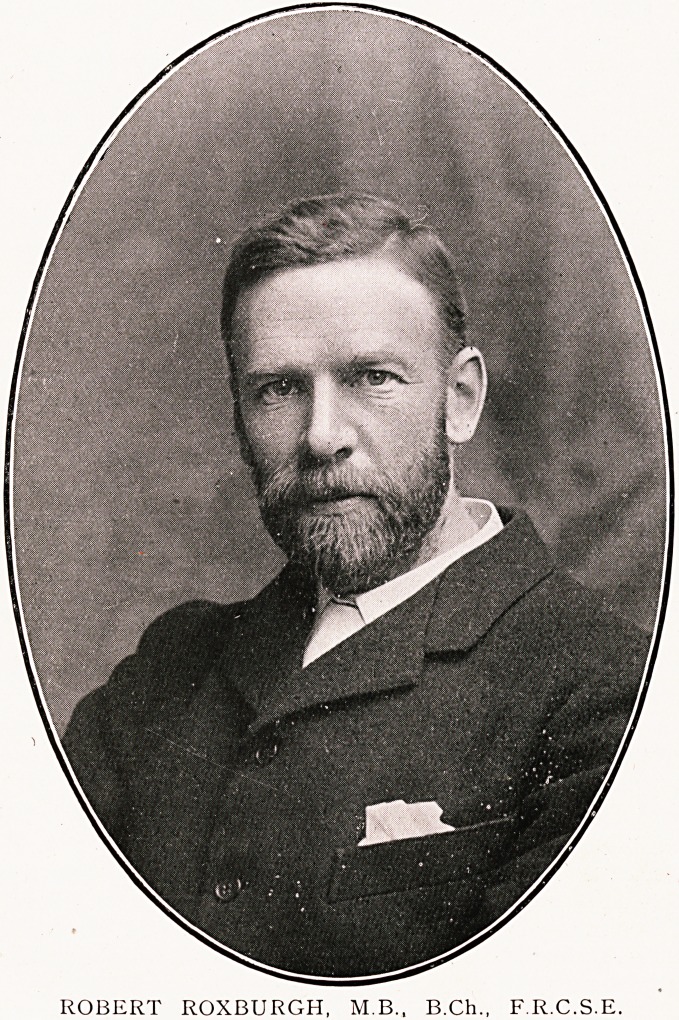# Robert Roxburgh, M.B., B.Ch., F.R.C.S.E.

**Published:** 1917-04

**Authors:** 


ROBERT ROXBURGH, M.B., B.Ch., F.R.C.S.E.

### Obituary.

### ROBERT ROXBURGH, M.B., B.Ch., F.R.C.S.E.

When Robert Roxburgh settled in Weston-super-Mare almost forty years ago, it was to succeed to the physician's practice of his brother-in-law, Dr. Frederick Gourlay, who had just then died. It was of a kind which made demands of no ordinary qualities, for Dr. Gourlay's magnetic personality had attracted patients who were extraordinarily responsive to the influence of a high and noble character.

To the comparatively young student, fresh from the Edinburgh training, and with added experience of the Vienna and Leipzig inspiring research work, it was a quite new experience to adjust himself to the new values of life disclosed in a physician's practice, and it was not without its possibilities of failure. How well and how courageously he adapted himself to novel conditions and new surroundings those of his friends who survive him can remember with admiration. The claims of his practice left him little time to continue pathological and bacteriological work in which he had been so keenly interested, and social demands—for was he not a welcome guest everywhere?—made great inroads on his leisure. Then, too, he threw himself with characteristic energy into every movement which had for its aim the welfare of his fellow-townsmen. Himself an accomplished musician, he helped its conductor to foster the local Philharmonic Society from insignificant beginnings into a well-ordered and most capable body of executants. And so with many other enterprises.

Dr. Roxburgh was President of the Bath and Bristol Branch of the British Medical Association in 1912.

His chief public work lay with the Weston-super-Mare Hospital. As Honorary Physician up to the time of his death, he achieved work of untold value. His out-patient room was always crowded because it was "his day," and although it was not incumbent on him to do this work, it was never hurried, and frequently he had only finished in time to hurry home to dinner After the commencement of the war, owing to the depletion of the hospital surgical staff, he gave his services as an anæsthetist at operations, frequently remaining until a late hour of the night.

As Consulting Physician to the Royal West of England Sanatorium, he found more work whenever his ripened skill was needed. It was always work with very little real leisure. His holidays were usually yachting cruises off the coast and in the lochs of his beloved Scotland. On these occasions his invitations were to congenial friends to join his family party.

About four years ago he lost his wife after a serious illness. He never seemed to be the same afterwards. Latterly his friends noticed the tired and worn expression he wore. It was clear to many that he was over-working, and at last the breakdown came, and after a short interval the end.

The writer remembers how he loved to speak of the happy days and experiences at Edinburgh, and of the distinguished members of his profession with or under whom he had studied. Lister was his hero ; he had at one time been his dresser, and many were the interesting experiences he would recount.

The loss to Weston-super-Mare by Dr. Roxburgh's death at the age of 63 is great. He was a wise physician, a true friend, and a man who thought and acted as one to whom all things that were pure and lovely and of good report were no mean ideals for the standard of a life to be well lived.

## Figures and Tables

**Figure f1:**